# Contrast analysis in ultrafast ultrasound blood flow imaging of jugular vein

**DOI:** 10.1007/s10396-023-01289-9

**Published:** 2023-02-09

**Authors:** Masaaki Omura, Kunimasa Yagi, Ryo Nagaoka, Hideyuki Hasegawa

**Affiliations:** 1 Faculty of Engineering, University of Toyama, 3190 Gofuku, Toyama, 93008555 Japan; 2 School of Medicine, Kanazawa Medical University, 1-1 Daigaku, Uchinada, Kahoku, Ishikawa 9200293 Japan

**Keywords:** Ultrafast ultrasound imaging, Clutter filter, Contrast map, Jugular vein, Erythrocyte deformation

## Abstract

**Purpose:**

The contrasts of flowing blood in in vitro experiments using porcine blood and in vivo measurements of human jugular veins were analyzed to demonstrate that the hemorheological property was dependent on the shear rate.

**Methods:**

Blood samples (45% hematocrit) suspended in saline or plasma were compared with examine the difference in viscoelasticity. Ultrafast plane-wave imaging at an ultrasonic center frequency of 7.5 MHz was performed on different steady flows in a graphite-agar phantom. Also, in vivo measurement was performed in young, healthy subjects and patients with diabetes. A spatiotemporal matrix of beamformed radio-frequency data was used for the singular value decomposition (SVD) clutter filter. The clutter-filtered B-mode image was calculated as the amplitude envelope normalized at the first frame in the diastolic phase to evaluate contrast. The shear rate was estimated as the velocity gradient perpendicular to the lateral axis.

**Results:**

Although nonaggregated erythrocytes at a high shear rate exhibited a low echogenicity, the echogenicity in the plasma sample overall increased due to erythrocyte aggregation at a low shear rate. In addition, the frequency of detection of specular components, defined as components beyond twice the standard deviation of a contrast map obtained from a clutter-filtered B-mode image, increased in the porcine blood at a high shear rate and the venous blood in healthy subjects versus patients with diabetes.

**Conclusion:**

The possibility of characterizing hemorheological properties dependent on the shear rate and diabetes condition was indicated using ultrafast plane-wave imaging with an SVD-based clutter filter.

## Introduction

Vascular function and blood conditions are essential to evaluate the signs of lifestyle-related diseases, e.g., hypertension and dyslipidemia. Blood viscoelasticity has been noted as a hemodynamic factor for impaired vascular function [1]. Compared with arteries, veins are less affected by pressure from the heart, and the flow velocity is lower. Thus, erythrocyte aggregation and coagulation are formed readily in veins, such as a red thrombus [2].

The flow field of blood (velocity gradient, i.e., shear rate) is affected by the hemodynamic characteristics of blood [3, 4]. The main component of blood is erythrocytes, and the flow pattern depends on the behavior of erythrocytes. The interaction of erythrocytes with plasma proteins results in the formation of erythrocyte aggregates, which increases the flow resistance and thus increases the viscosity of the blood at a low shear rate. Erythrocytes with a high content of compounds contribute to reducing the flow resistance by accepting passive mechanical deformations, resulting in a decrease in blood viscosity at a high shear rate. On the other hand, as the erythrocyte deformation capacity decreases, the flow field is disturbed, and the blood viscoelasticity increases.

High-frequency ultrasound (> 20 MHz) has the potential for evaluating the erythrocyte aggregation size by analyzing the power and frequency dependence of the ultrasonic backscatter signals [5–9]. Because such studies also aimed to characterize erythrocytes under a slow velocity, such as that in a peripheral vein, conventional focused line-by-line imaging (several 10 to 100 Hz) was enough to observe moving erythrocytes at a low shear rate. An in vitro study has revealed the efficiency of high-frame-rate plane-wave imaging with 7.5 MHz ultrasound to characterize static tissue [10] and flowing erythrocytes in an agar phantom [11] in the absence of clutter signals. However, in the case of in vivo applications with such ultrasonic frequencies, a clutter filter is required to visualize echoes from erythrocytes by suppressing clutter signals from surrounding tissues.

A clutter filter with high-frame-rate imaging can improve the sensitivity of blood flow signals and provide more efficient discrimination from tissue signals. The tissue signals are commonly removed using a high-pass finite or infinite impulse response filter [12]. This process regards the low temporal frequency component as echoes from slowly moving tissues. Singular value decomposition (SVD) is an advanced technique for clutter filter design [13–16]. An SVD-based clutter filter has improved the sensitivity in clutter rejection and extraction of tiny vessels with slow velocities [17, 18].

In our pilot clinical study, the spatial pattern of blood flow signals was observed as a speckle pattern in a jugular vein owing to an SVD-based filter [19]. This study explored in detail the spatial features such as the contrast of ultrasonic echoes from flowing blood with a clutter filter to demonstrate that the hemorheological property was dependent on the shear rate. The spatial echogenicity of porcine blood in a tissue-mimicking phantom was investigated from low to high steady shear rates comparable with the human jugular vein [20]. In addition, point-like or streak-like regions along the flow direction were confirmed as hyperechoic components (not shown in detail to date). To investigate the underlying mechanism of such a phenomenon, the frequency of hyperechoic components was also evaluated by means of contrast analysis. Secondly, in vivo measurements in jugular veins were carried out to confirm the spatial echogenicity under physiological flow conditions. Erythrocyte backscattering was compared between young, healthy subjects and patients with diabetes in a cardiac cycle. The novelty of the present study was to identify not only functional (flow velocity), but also backscatter properties of blood with a higher penetration depth in a vasomotor center than high-frequency ultrasound.

## Materials and methods

### Blood sample and flow system

Whole porcine blood anticoagulated with sodium citrate was centrifuged at room temperature (24 ℃) within 24 h of its collection, as described in previous studies [11, 21, 22]. Briefly, whole blood was centrifuged (4000 g for 10 min) to separate erythrocytes, buffy coat, and plasma. Erythrocytes were washed twice with phosphate-buffered saline (PBS). Plasma was also centrifuged (15,000 g for 30 min) to create platelet-poor plasma. The buffy coat was removed, and the hematocrit was adjusted to 45% in PBS or autologous plasma to compare the difference in viscoelasticity of the solution.

**Fig. 1:**
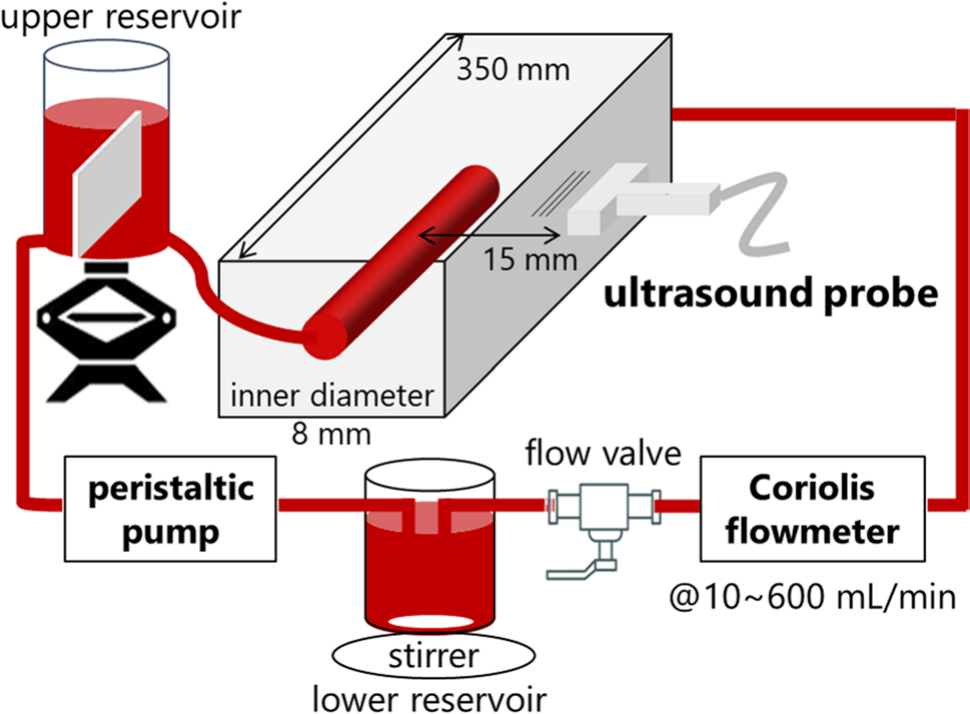
Diagram illustrating the experimental setup for the ultrasonic backscatter measurements in porcine blood under steady laminar flow

A blood sample of 0.8 L was circulated in a steady flow system, as shown in Fig. 1. The blood sample was pumped from the lower to the upper reservoir using a peristaltic pump (Model 07,528–10, Masterflex). A magnetic stirrer in the lower reservoir prevented erythrocytes from settling. The partition was placed in the upper reservoir to block the flow disturbance and inflow of air bubbles. The blood was passed through the wall-less lumen (8 mm in diameter and 350 mm in length) in a tissue-mimicking phantom composed of 2 wt% agar, 7 wt% graphite, and 91 wt% degassed water. The distance from the phantom surface to the center lumen was 15 mm. The speed of sound and attenuation coefficient of the phantom were 1530 m/s and 0.70 dB/cm/MHz, respectively. The steady flow was controlled with a flow regulator (CY-1208, ASOH) and monitored with a Coriolis flowmeter (FD-SF8, Keyence). Ultrasound data were obtained at flow rates from 10 to 600 mL/min.

### In vivo* measurement*

All procedures were performed according to the ethical standards of the responsible committee on human experimentation and with the Helsinki Declaration of 1964 and later versions. The Ethics Committee of Toyama University Hospital approved the study protocol (IRB# R2019135). Web notifications informed all participants that they could opt out at any time. Informed consent was obtained from all participants.

The diagnoses of type 2 diabetes were based on the American Diabetes Association’s diagnostic criteria; diagnosed using HbA1c ≥ 6.5% (National Glycohemoglobin Standardization Program), a fasting blood glucose concentration of ≥ 126 (7.0 mol/L) mg/dL, or a random blood glucose concentration of ≥ 200 mg/dL [23], or if the health questionnaire indicated current medications for diabetes.

The jugular veins of young, healthy subjects ($n$=5, 21–29 y.o., male) and patients with diabetes ($n$=5, 55–88 y.o., male) were scanned in vivo. Healthy subjects and patients with diabetes were measured at intervals of 3 and 12 h after a meal. All subjects were in the supine position and breathed normally during the measurement.

### Data acquisition

A 7.5 MHz linear array probe (UST-5412, Fujifilm) was placed in the middle of the tissue-mimicking phantom at the maximum diameter of the lumen and along the long axis of the human vein. A single plane wave at an angle of 0° was transmitted in each frame at pulse repetition periods of 200 µs (porcine blood experiment) and 480 µs (in vivo measurement). Tukey apodization with a coefficient of 0.4 was used in transmission. The RF channel data were acquired at a sampling frequency of 31.25 MHz for 0.5 s (porcine blood experiment) and 0.96 s (in vivo measurement) using a research-platform scanner (RSYS0016, Microsonic). Offline beamforming was carried out using the delay-and-sum method with the dynamic aperture (F-number of 1) [24]. The beamformed RF data were created on a pixel-by-pixel basis (depth 25 µm × lateral 100 µm)　at a speed of sound of 1540 m/s.

### Clutter filtering and contrast analysis

First, a spatiotemporal filter based on SVD was applied to the beamformed RF data to emphasize the blood flow signal [17]. Briefly, a spatiotemporal matrix [temporal 2500 (porcine blood experiment) or 2000 (in vivo measurement) frames] composed of the beamformed signal in all the frames and was used for the SVD filter. The low- and high-rank threshold of singular values for clutter, blood flow, and system noise components were empirically unified among the porcine blood experiments (from − 60 to − 30 dB) and in vivo (from − 60 to − 34 dB) measurements, respectively.

The filtered RF data were transformed to the amplitude envelope using the Hilbert transform. Ensemble averaging was applied in the amplitude envelope for 4 frames. The contrast map was calculated by normalizing the amplitude envelope as

1 $$contrast=10{\mathrm{log}}_{10}\left[\frac{\mu (z,x,i)}{{\mu }_{total}}\right],$$

where $\mu$ and ${\mu }_{total}$ are the mean of the power of the amplitude envelope in each local region (depth $z,$ lateral $x$) at typical frame $i$ and the whole lumen (in the first frame in diastolic phase in vivo). The lumen of the vein was manually segmented at the systolic phase. The kernel size was 1.0 × 1.0 mm^2^. To detect hyperechoic components in blood flow images, the threshold was empirically calculated as $E[contrast]$ + $2\times SD$, where $E$[・] and $SD$ were the mean and standard deviation of the contrast for all frames. The region detection was evaluated as the pixel area ratio of hyperechoic components and the whole lumen.

### Flow analysis

Block-matching analysis was carried out to calculate the blood flow velocity. The block size was 60 × 60 pixels (1.4 × 6.0 mm^2^ for the depth and lateral directions), and the search distance in both directions was 40 pixels in reference to our previous simulation study on blood mimicking fluid measurement [25, 26]. Adjacent macro blocks overlapped each other by 0.5 and 1.0 mm in the depth and lateral directions, respectively. The normalized cross-correlation function was up-sampled using the reconstructive interpolation method to estimate subsample displacement [27, 28]. Both the lateral and axial interpolation factors were 4. Also, ensemble averaging of the correlation function was performed for 2 ms (4 frames).

The spatial slope of the flow velocity profile, i.e., shear rate, was calculated in the neighborhood around the proximal and distal boundaries between the vein wall and lumen, which were manually selected. A gate length of 3 mm was set from each boundary in each beam line to estimate the slope using the least-squares method.

## Results

**Fig. 2:**
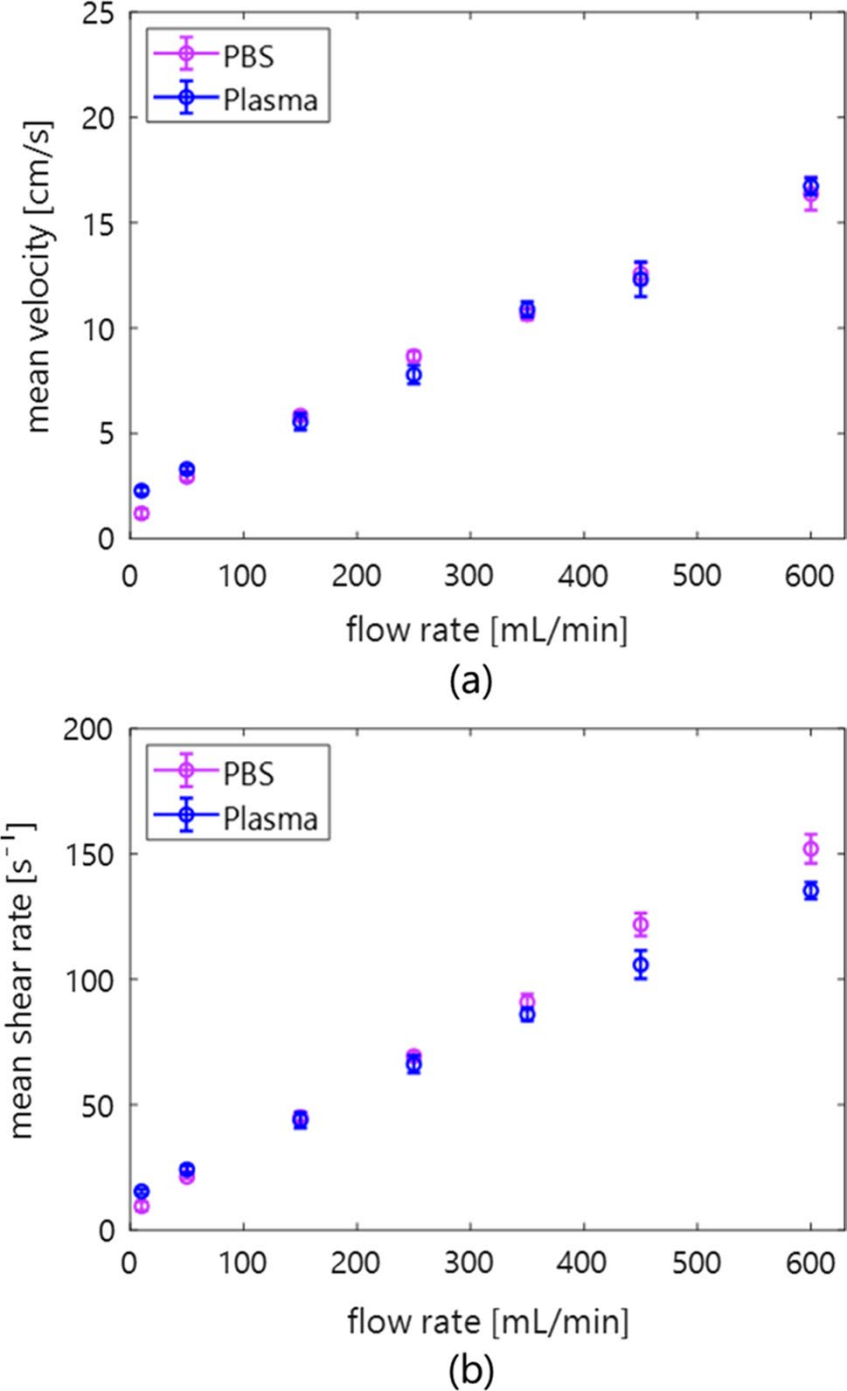
Mean and standard deviation of velocity (**a**) and shear rate (**b**) in the porcine measurement as a function of the flow rate

Figures 2(a) and (b) show the relationship among flow rate, mean velocity, and shear rate in PBS and plasma samples. Each mean shear rate was calculated as the spatiotemporal mean of those estimated at the proximal and distal boundaries. Both mean velocity and shear rate linearly increased with the increase of the flow rate.

**Fig. 3:**
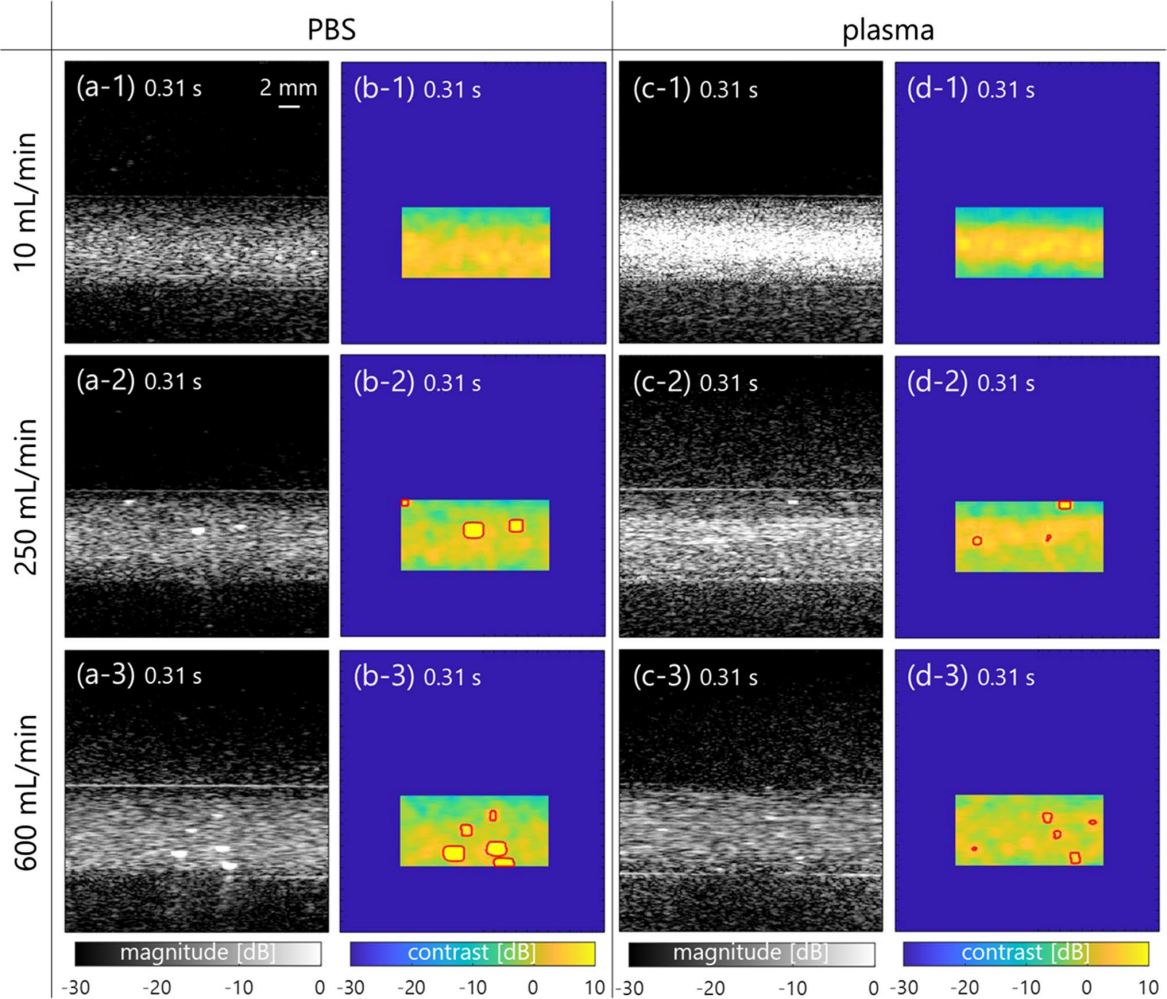
B-mode and contrast images of PBS [(**a**) and (**b**)] and plasma [(**c**) and (**d**)] samples at the typical time at each flow rate [(1)–(3)]. The region detection is enclosed by a red line

**Fig. 4:**
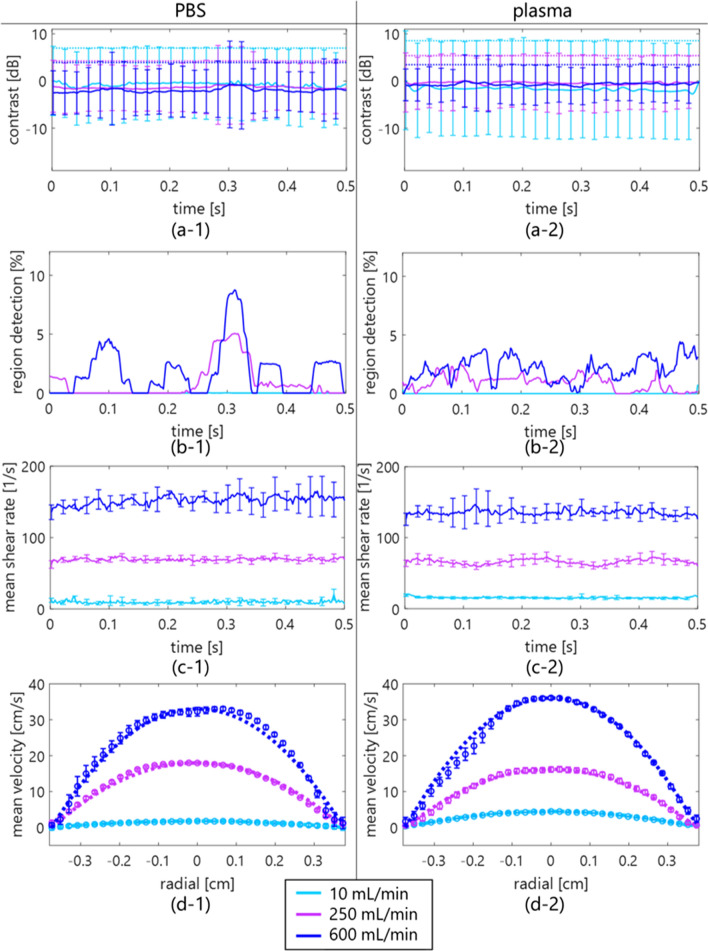
Temporal variations in contrast (**a**), region detection (**b**), and shear rate (**c**) in the porcine blood experiment. The dotted line in the contrast plot shows the threshold estimated by mean and standard deviation of contrast in all frames. Each marker (mean) with solid and dotted lines (curve fitting) and the error bar (standard deviation) are shown in the radial velocity profile (**d**)

Examples of B-mode and contrast images for detection of hyperechoic regions (enclosed by a red line) in PBS and plasma samples are displayed in Fig. 3. These images were normalized by the same amplitude envelope value and shown with the same grayscale range. Although the echo intensity of the plasma sample is higher than that of the PBS at a low flow rate, i.e., low shear rate, those of both samples are similar at a high shear rate. Moreover, a hyperechoic component enclosed by a red line was locally distributed in both samples at flow rates of 250 and 600 mL/min. Figures 4(a)-(c) show the temporal variations of contrast, hyperechoic region detection, and shear rate among three flow rates. The threshold for hyperechoic region detection was also plotted with the dotted line in Fig. 4(a). The higher the shear rate is, the more frequently the hyperechoic region detection irregularly appears. The error bars ($2SD$) of contrast exceeded the threshold at the time with occurrences of hyperechoic region detections. Also, Fig. 4(d) shows the radial velocity profile of both samples at the three flow rates. The marker and error bar are the temporal mean and standard deviation, respectively, at each flow rate or radial position. The velocity profiles were parabolic in both samples regardless of flow rates. Note that the result of curve fitting was from $n$=1.99 (minimum) to 2.03 (maximum) in $v\left(r\right)={v}_{\mathrm{max}}[1-{(r/R)}^{n}]$, where $v$ was the velocity at radial distance $r$ from the central axis in the lumen radius $R$ to confirm the laminar flow ($n=2.00$).

**Fig. 5:**
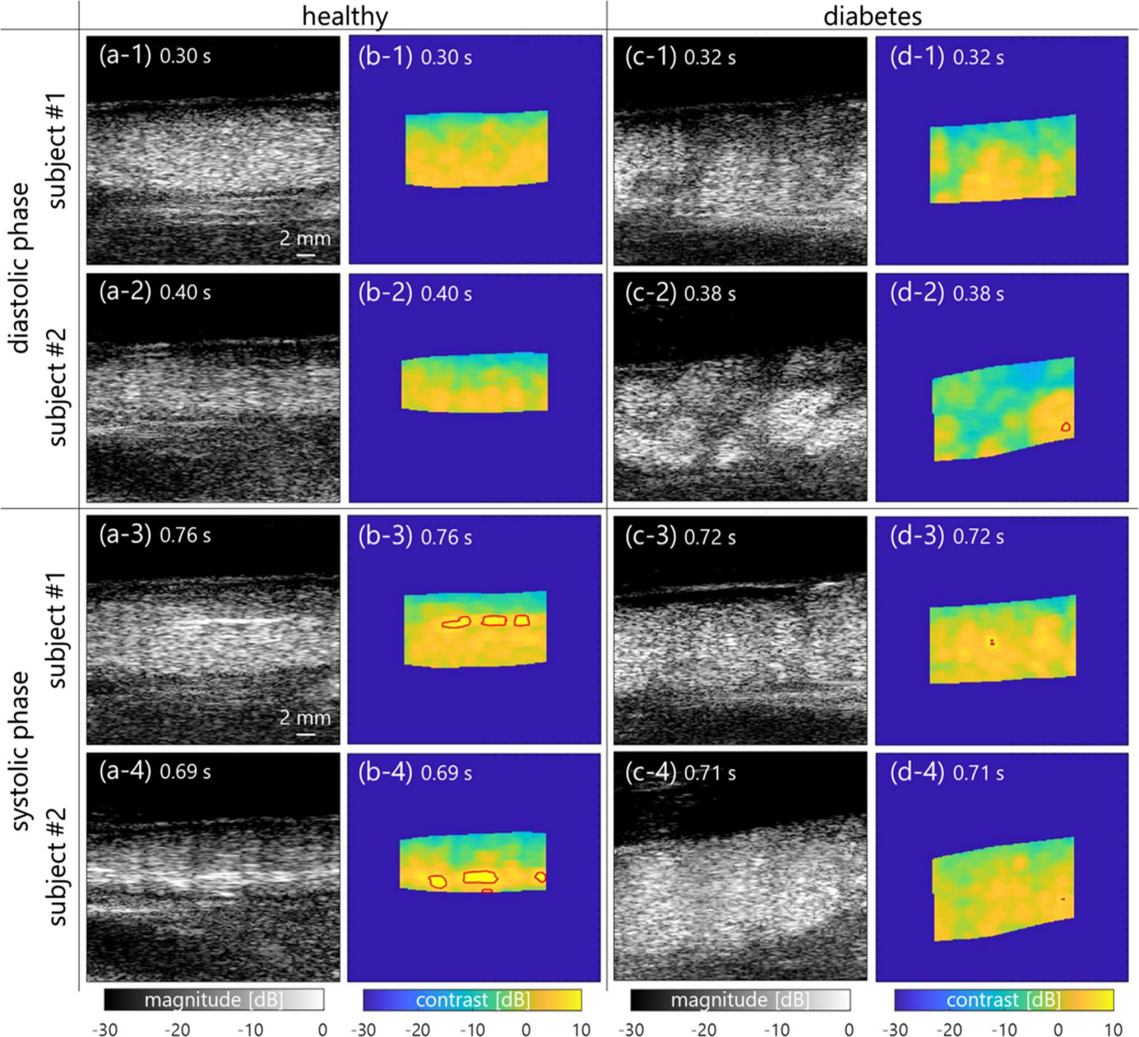
B-mode and contrast images of young, healthy subjects [(**a**) and (**b**)] and patients with diabetes [(**c**) and (**d**)] at the diastolic (from 0 to 0.5 s) [(**1**) and (**2**)] and systolic (from 0.5 to 0.96 s) [(**3**) and (**4**)] cyclic phases

**Fig. 6:**
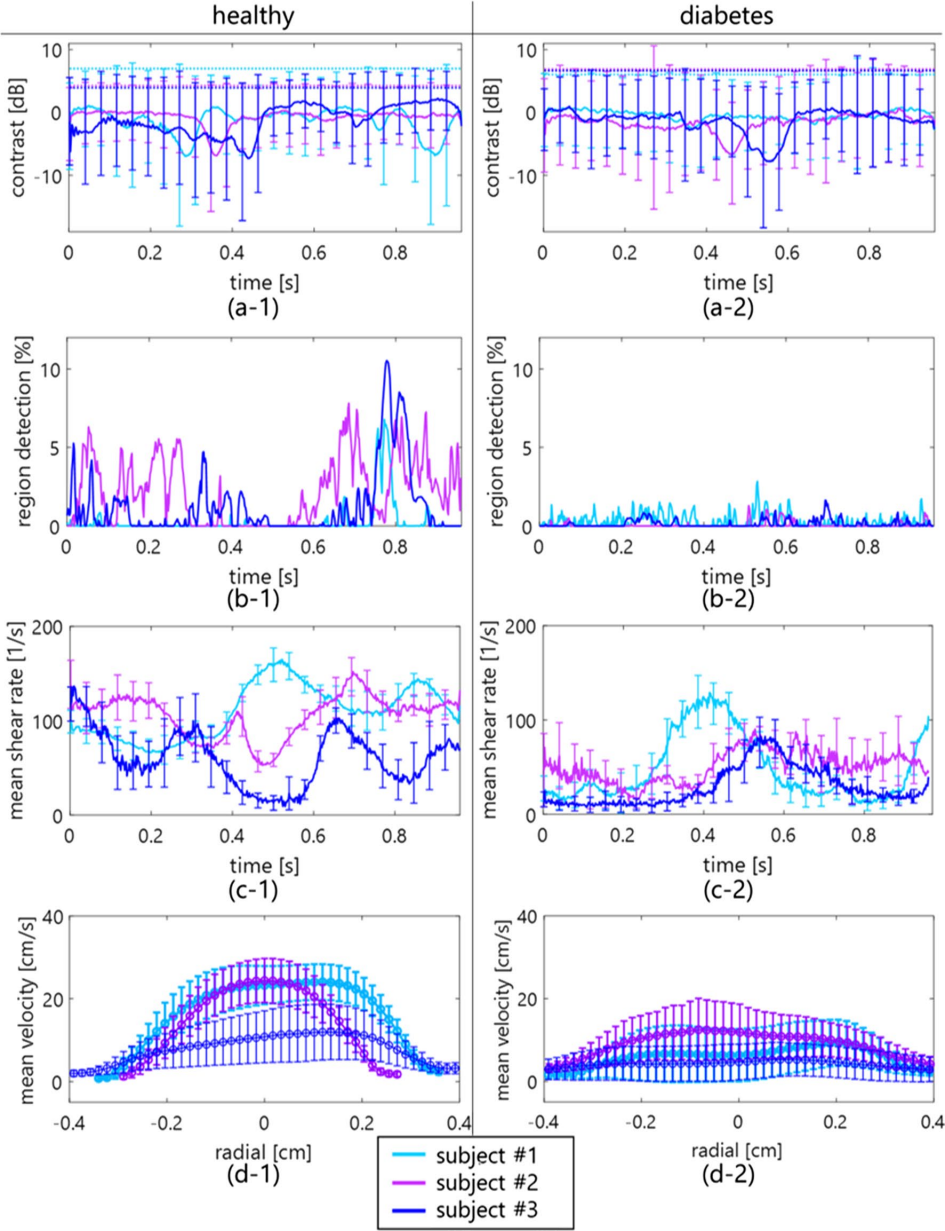
Temporal variations in contrast (**a**), region detection (**b**), and shear rate (**c**) in young, healthy subjects and patients with diabetes. The radial velocity profiles in the subjects are also shown in (**d**). Each line and error bar shows the mean and standard deviation of each spatial or temporal feature

As visualized in Fig. 5, B-mode and contrast images with hyperechoic region detection in in vivo measurements are compared between young, healthy subjects and patients with diabetes. The texture in the contrast image was more homogeneous in the healthy subjects than in the patients with diabetes at the former phase (from 0 to 0.5 s) in Figs. 5(1) and 5 (2). In contrast, the hyperechoic regions were frequently confirmed in the healthy subjects at the latter cyclic phase (from 0.5 to 0.96 s) in Figs. 5(3) and 5 (4). Figures 6(a)-(c) also show the temporal variations of contrast, hyperechoic region detection, and shear rate of healthy subjects and patients with diabetes. Although the hyperechoic region detection in the healthy subjects was irregularly distributed around 5–10% at the maximum, that in the patients with diabetes was less than 1%. The mean shear rate fluctuated more in the healthy subjects in comparison to the patients with diabetes. Figure 6(d) shows the mean velocity at each radial position in the example of healthy subjects and patients with diabetes. The profile of the mean velocity was close to parabolic in the healthy subjects. However, it was complex and its mean value was lower in patients with diabetes.

**Fig. 7:**
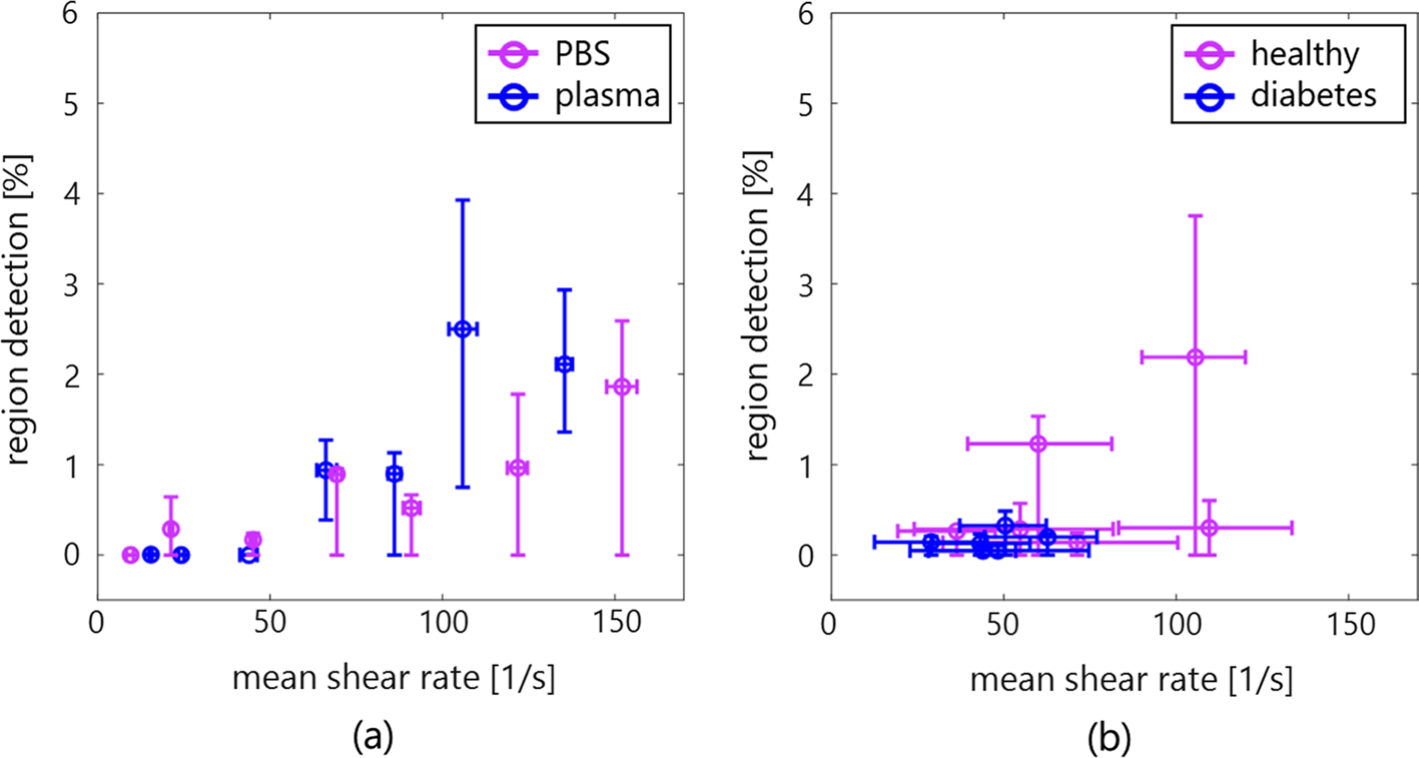
Comparison of mean region detection and shear rate in the porcine blood (**a**) and in vivo measurements (**b**). Each error bar shows the interquartile range

Figure 7(a) compares the temporal mean of hyperechoic region detection with the shear rates in PBS and plasma samples. The hyperechoic region detection was beyond 0% over around 50 s^−1^ in both samples. Furthermore, the mean shear rates were partly higher than 50 s^−1^ (four by five subjects) with much greater frequencies of hyperechoic region detection (three by five subjects) beyond the mean of patients with diabetes, as expressed in Fig. 7(b).

## Discussion

This study investigated the spatial feature of blood flow in porcine blood and the in vivo jugular vein of young, healthy subjects and patients with diabetes using an SVD clutter filter. In the porcine blood experiment, the properties of erythrocyte rheology such as aggregation were confirmed to be different at a low shear rate (around < 50 s^−1^) between PBS and plasma samples, as shown in Figs. 3(a-1) and 3(c-1). Erythrocyte aggregation caused by the force between erythrocytes and protein in the plasma overall induced increasing echogenicity, consistent with previous studies [11, 21, 29, 30]. However, erythrocyte disaggregation caused by a high shear rate (> 50 s^−1^) was visualized as lower echogenicity in both samples in Figs. 3(a-3) and (c-3). These findings were consistent with the previous studies about the dependence of ultrasonic backscatter power on the shear rate and erythrocyte rheology [11, 21, 29, 30]. In addition, high echogenicity components with the shape of the point spread function or streak-like shape extending along the flow direction appeared in the B-mode image in Figs. 3(a-2)–(a-3) and Figs. 3(c-2)–(c-3), and the frequency of hyperechoic region detection was higher at a high shear rate than at a low shear rate (several 50 s^−1^), as expressed in Fig. 7(a).

There is a possibility that the high echogenicity component arises from the anisotropic features, e.g., erythrocyte deformation and alignment, against plane-wave transmission and reception at a high shear rate. When ultrasound is insonified to a muscle from a direction perpendicular to the fiber orientation, the backscatter power is naturally higher than the case of insonification along the fiber orientation [11, 31]. Similarly, the high echogenicity component will appear when erythrocytes are extended and aligned along the flow axis, which would be caused by the force across the lumen diameter. The property of erythrocyte deformation depends on hematocrit (about > 20%) and the viscoelasticity of erythrocytes and plasma [32]. The viscosity of the sample is considered to be different between PBS and plasma samples, while high echogenicity components are present in both samples because of the same viscoelasticity of the erythrocyte membrane. Although these features were also confirmed at the typical frames in the jugular vein of the young, healthy subjects, B-mode textures were inhomogeneous [in Figs. 5(c-1)–(c-2)] due to the flow turbulence [in Fig. 6(d-2)] in the patients with diabetes. The soft and young erythrocytes with deoxyhemoglobin in the healthy subjects have a high capacity for deformation in response to the shear rate [33]. The higher mean and variation of shear rates in healthy subjects [Figs. 6(c-1) and 7 (a)] indicate the prompt response of the erythrocyte deformability. Conversely, a chronic lower shear rate and irregular velocity profile in aging patients with diabetes [Figs. 6(c-2) and 7(b)] might be distinct, with less deformability and irregular alignment of the hard erythrocyte membrane. It is known that diabetes results in various changes in the factors of the erythrocyte membrane and interior composition [34, 35]. These factors are related to the functional characteristics of the erythrocyte s, which can include impairment of the deformability. Although this hypothesis needs to be proven for less fluidity of erythrocyte membrane with other techniques such as optical observation under microcirculation, this is consistent with the suggestions in previous studies [36, 37].

The hyperechoic component was observed at both the near wall and central lumen at the same time in Figs. 3 and 5. In the present study, the shear rate was used as an index describing the flow field to discuss the possibility of the contribution of erythrocyte deformation and alignment contributing to the hyperechoic region. However, the temporal and spatial changes in echogenicity under steady and pulsatile flows cannot be explained by shear rate alone. It has been hypothesized that local acceleration and velocity are other factors that induce the change in hemorheological properties [37, 38] because several instances of changes in the contrast occur around the systolic phase of the flow. In addition, it would be possible for each erythrocyte to move independently without aggregation. In such a case, the major axes of erythrocytes might be aligned with the flow axis at a high shear rate. Because erythrocytes can rotate in the narrow lumen [39], each erythrocyte may rotate once over an oscillatory cycle in such a way that the long biconcave axis may align with the flow direction during acceleration. Hence, the mechanism of appearance of the hyperechoic component must be considered with the spatial flow velocity profile during acceleration.

The shape of the hyperechoic component seemed to be different between porcine blood (similar shape but larger size as compared to the point spread function in Fig. 3) and in vivo experiments (streak-like shape in Fig. 5). Although experimental data were obtained under steady flow, as shown in Fig. 4, in vivo data were obtained under pulsatile flow in Fig. 6. While the shear rate and pressure fluctuations are more rapid in vivo, the erythrocytes are more likely to be subjected to external physical forces. The acceleration and deceleration of the flow might enhance rouleaux formation because there are more chances to form a larger rouleaux when the flow is accelerating [40, 41]. Hence, the streak-like pattern presumably caused by erythrocyte deformation might be more evident in in vivo data. The spatial feature of erythrocytes under pulsatile flow is not fully understood, especially in noninvasive ultrasound imaging. It will be necessary to reproduce the pulsatile flow in experimental data in a future study.

Beam-steering transmission and reception in plane-wave imaging at a tilted angle of flow would also be useful to explain the anisotropic features because their alignment and deformability across the lumen diameter induce angle dependence of the echogenicity variation [37]. In the present study, the measurement angle between the propagation direction of the incident wave and vein wall was preferably perpendicular in the porcine blood and in vivo measurements. In addition, the spread of the transmit beam may affect the length of the streak-like pattern. Such an effect of a transmit beam will be investigated in a future study using high-frame-rate imaging with plane waves and a focused beam [42, 43].

Another possible explanation for the high echogenicity components, particularly with the shape of the point spread function, is platelets in the porcine blood experiment. A previous study using porcine blood mentioned that platelet aggregates were echogenic at a high shear rate (around 300 s^−1^) [22]. The present study was also conducted with high-speed centrifugation to produce platelet-poor plasma. However, quantitative measurement of the number of platelets was not performed. The amount of platelets needs to be confirmed in the blood sample test.

The shear rate estimates must be influenced if flow profiles are nonparabolic, such as flow turbulence. In this study, the shear rate around the boundaries between the vein wall and lumen was calculated by estimating the spatial gradient of the flow velocity using the least-squares method. As shown in Figs. 2 and 4, the shear rate was stably calculated in the laminar flow with the parabolic profile. However, features in B-mode images were inhomogeneous [in Figs. 5(c-1)–(c-2)] due to the irregular velocity profile due to flow turbulence [in Fig. 6(d-2)], especially in patients with diabetes. Another index such as vorticity might be beneficial to understand the relationship between spatial features of echogenicity and flow pattern.

The contrast map was created to normalize the base amplitude envelope among subjects due to the different attenuation through tissues and absolute backscatter from blood dependent on the hematocrit. The outliers, i.e., region detection, were empirically defined beyond $E[contrast]$ + $2SD$ based on the contrast in all frames. The high echogenicity components beyond the threshold could be detected in the porcine experiment at a high shear rate of PBS and plasma samples, as shown in Fig. 7(a). Although these components were frequently detected in the healthy subjects in Fig. 6(b-1), the pulsatile flow was shown as the fluctuations of velocity and shear rate in Fig. 6(c-1). The cyclic change of contrast should be dependent on the cutoff property of the clutter filter. As shown in Figs. 6(a-1) and 6 (a-2), the minimum contrast was around − 8 to − 6 dB at a low shear rate of around 10–60 s^−1^ because the discrimination between clutter and blood flow signals might be difficult under low shear rate (velocity) conditions. This study empirically determined the threshold based on the contrast in all frames, so the performance of hyperechoic region detection would be poor, resulting in lower contrast resolution. Hence, it is necessary to consider a pulsatile flow case in the porcine blood experiment with tissue motion. High-frequency ultrasound (> 20 MHz) with high-frame-rate imaging is one of the solutions to enhance the backscatter power from blood flow without a clutter filter. Also, the cyclic change in the region detection should be dependent on the thresholding process of contrast. Further development is necessary to migrate other spatial features such as ultrasonic envelope statistics [44, 45] and high-order moments. In addition, a robust and better clutter filter scheme using channel domain implementation and spatial singular value vectors would be useful in future studies for ultrasonic backscatter from blood flow.

## Conclusion

The spatial feature of ultrasonic backscatter from erythrocytes dependent on the shear rate was confirmed in porcine blood and in vivo jugular vein measurements. Hemorheological properties such as aggregation, association, and anisotropy were shown in the amplitude envelope through an SVD-based filter. The high echogenicity regions beyond twice the standard deviation of the contrast map could be detected in the porcine blood at a steady high shear rate and in young, healthy subjects in comparison to patients with diabetes.

## Data Availability

The datasets generated during and analysed during the current study are available from the corresponding author on reasonable request.
