# Correction to: Contrast analysis in ultrafast ultrasound blood flow imaging of jugular vein

**DOI:** 10.1007/s10396-024-01441-z

**Published:** 2024-03-23

**Authors:** Masaaki Omura, Kunimasa Yagi, Ryo Nagaoka, Hideyuki Hasegawa

**Affiliations:** 1 Faculty of Engineering, University of Toyama, 3190 Gofuku, Toyama, 93008555 Japan; 2 School of Medicine, Kanazawa Medical University, 1-1 Daigaku, Uchinada, Kahoku, Ishikawa 9200293 Japan

**Correction to: Journal of Medical Ultrasonics (2023) 50:131–141** 10.1007/s10396-023-01289-9

The article “Contrast analysis in ultrafast ultrasound blood flow imaging of jugular Vein”, written by Masaaki Omura, Kunimasa Yagi, Ryo Nagaoka and Hideyuki Hasegawa, was originally published Online First without Open Access. After publication in volume 50, issue 2, page 131–141 the author decided to opt for Open Choice and to make the article an Open Access publication. Therefore, the copyright of the article has been changed to © The Author(s) 2023 and the article is forthwith distributed under the terms of the Creative Commons Attribution 4.0 International License, which permits use, sharing, adaptation, distribution and reproduction in any medium or format, as long as you give appropriate credit to the original author(s) and the source, provide a link to the Creative Commons licence, and indicate if changes were made. The images or other third party material in this article are included in the article’s Creative Commons licence, unless indicated otherwise in a credit line to the material. If material is not included in the article’s Creative Commons licence and your intended use is not permitted by statutory regulation or exceeds the permitted use, you will need to obtain permission directly from the copyright holder. To view a copy of this licence, visit http://creativecommons.org/licenses/by/4.0.

The original article has been corrected.

